# The 7-Repeat (7R) Variant of the *DRD4* Gene and Ways of Coping with Stress in Medical Professionals—A Preliminary Study

**DOI:** 10.3390/ijms26178653

**Published:** 2025-09-05

**Authors:** Katarzyna Bliźniewska-Kowalska, Piotr Gałecki, Piotr Czarny, Janusz Szemraj, Łukasz Kołodziej, Małgorzata Gałecka

**Affiliations:** 1 Department of Adult Psychiatry, Medical University of Lodz, 91-229 Lodz, Poland; piotr.galecki@umed.lodz.pl; 2 Department of Medical Biochemistry, Medical University of Lodz, 92-215 Lodz, Poland; piotr.czarny@umed.lodz.pl (P.C.); janusz.szemraj@umed.lodz.pl (J.S.); 3 Laboratory of Medical Genetics, Faculty of Biology and Environmental Protection, University of Lodz, 90-236 Lodz, Poland; lukasz.kolodziej@edu.uni.lodz.pl; 4 Bio-Med-Chem Doctoral School of the University of Lodz and Lodz Institutes of the Polish Academy of Sciences, 90-237 Lodz, Poland; 5 Department of Psychotherapy, Medical University of Lodz, 91-229 Lodz, Poland; malgorzata.galecka@umed.lodz.pl

**Keywords:** *DRD4*, health care professionals, coping with stress

## Abstract

How we deal with stress and certain traits of our personality can be influenced not only by psycho-social factors, but also by biological factors, including genetics. Research studies suggest that the long allele of the *DRD4* gene (*DRD4*-7R) is associated with novelty seeking and risk taking—features potentially important for healthcare workers. The aim of this study was to assess the *DRD4*-7R polymorphism and its impact on propensity for risk taking and ways of coping with stress in medical professionals This preliminary study involved 82 volunteers from among active healthcare professionals, including 33 medical doctors (MDs). All participants were asked to fill out psychological questionnaires for assessment of their stress-coping strategies (Mini-COPE) and risk-taking propensity (IVE). A swab of the inside of the cheek was taken from the study participants to determine the polymorphism within the gene for the dopamine D4 receptor gene (*DRD4*). In our study, medical doctors (MDs) tend to have the *DRD4*-7R allele more often than other medical professionals. Our study also indicates that *DRD4*-R7 allele carriers are significantly less likely to use stimulants and other substances to help themselves with stress coping and less likely to expect emotional support. The *DRD4* gene polymorphism may be important for the development of specific personality traits and ways of coping with stress. However, further research with larger numbers of participants is needed.

## 1. Introduction

Due to their high exposure to stress in their professional lives, healthcare workers constitute an interesting research group in the context of studies on methods of coping with stress, as well as the psychological and biological determinants of stress responses.

Working in the health professions involves a great deal of responsibility and, consequently, exposure to stress. Furthermore, staff shortages and working under time pressure are associated with high overloads for healthcare workers. A significant phenomenon among occupations that require intensive contact with people is burnout syndrome. It occurs under the influence of prolonged and poorly managed stress [1], in which the employee feels chronically overtired and dissatisfied with the job, and becomes distant and less and less committed to work [2,3]. Although occupational burnout is not recognized as a disease by the WHO in the ICD-11 classification of diseases, it is a serious medical and social problem that is a response to chronic occupational stress [4]. Hence, it seems very important to assess the severity of stress and coping methods among medics.

It is often said that medical professionals experience high levels of stress, but it should be noted that environmental factors are not the only ones that play a role here. How we deal with stress and certain personality traits can be influenced not only by psycho-social factors, but also by biological factors, including genetics. Studies described the association of the *DRD4* (Dopamine Receptor D4) gene with temperament or personality traits [5,6,7]. There are several types of polymorphisms involving the *DRD4* gene. The 48-base pair variable number tandem repeat (VNTR) in exon 3 of the *DRD4* gene in humans ranges from 2 to 11 repeats [8]. The so-called “Nomad gene” explored in this study is *DRD4*, with seven repeats (*DRD4*-7R). The name comes from a population of wanderers, the Nomads, who have higher frequencies of 7R alleles than the ones in sedentary tribes [9]. Research suggests that the long allele (seven or more repeats) is associated with novelty seeking and risk taking, and limited emotional responses, but it is also associated with retained attention processing of emotional stimuli and effective problem solving [10,11]. These are very important traits in medical professions.

It should also be noted that people working in healthcare are often characterized by a high level of ambition and a strong drive to achieve difficult and important goals. A large meta-analysis focusing on profiling non-cognitive features, i.e., personality traits, behavioral patterns and emotional intelligence, of medical professionals revealed many shared characteristics (homogeneity) among different representatives of this professional group. All medical professions exhibit personality traits such as agreeableness, cooperativeness and independence, as well as lower levels of neuroticism [12].

Due to the high level of work-related stress among healthcare workers and the specific nature of this professional group, it seems reasonable to investigate the associations between certain temperament traits (impulsivity, risk taking), stress-coping strategies, type of work performed, and biological factors. In general, research into the relationship between gene polymorphism and psychological traits is important because it allows researchers to better understand the basis of our individuality.

Given the abovementioned features, the ‘Nomad gene’, i.e., the long allele (more than seven repeats) of the *DRD4* gene, seems to be an ideal candidate for this type of research. The polymorphism of this gene has already been studied among investors [13] and Senegalese fishermen [14], but never among healthcare workers.

The aim of this study was to assess the *DRD4*-7R polymorphism and its impact on propensity for risk taking and ways of coping with stress in medical professionals.

## 2. Results

### 2.1. Genetic DRD4-7R Results

The detailed characteristics of the *DRD4* gene polymorphism in the study group are shown in Table 1. The 4R allele is the most common. Only about 1/3 of participants had at least one *DRD4*-7R allele (Table 1 and Table 2). Therefore, the study group was divided into those who are carriers of at least one long allele (7R) and the others. The occurrence of 7R alleles was tested by gender (*p* = 0.5072), age group (*p* = 0.9766), profession (*p* = 0.0067), seniority (*p* = 0.9198), and type of work position (surgical, non-surgical and emergency/IC) (*p* = 0.5471). The study cohort was represented by the following professions: medical doctors (MDs) (*n*= 33), nurses (*n* = 37), paramedics (*n* = 4), physiotherapists (*n* = 3), clinical psychologists (*n* = 4), and a midwife (*n* = 1).

The results showed that medical doctors (MDs) were more likely to have *DRD4*-7R allele than other medical professions (Table 3). Table 4 details the breakdown into individual professional groups, additionally distinguishing a relatively well-represented group of nurses (*n* = 37). There was also a statistically significant difference between the groups (Kruskal–Wallis test), but post hoc tests did not detect any significant differences. On the other hand, a homozygote carrying two *DRD4*-7R alleles was found in two non-MD individuals—a clinical psychologist and a nurse.

Logistic regression estimates OR = 3.70 (95% CI: 1.44–9.52) (*p* = 0.0067). The interpretation reads that the odds of possessing the investigated 7R allele were 370% higher among the studied medical doctors (MDs) as compared to their non-MD counterparts.

### 2.2. Risk Taking and Impulsivity—IVE Test Results

Comparisons between groups (female and male, medical doctors (MD) and other health professionals, 7R allele carriers and others) with regard to the IVE test results were carried out. Statistical analysis showed that male respondents were statistically significantly more willing to take risks (venturesomeness) than females (*p* = 0.001). In contrast, female respondents were characterized by greater empathy (*p* = 0.008) (Table 5). Medical doctors (MDs) were more likely to take risks than other healthcare professionals (Table 6). In addition, the analysis was supplemented with a comparison of occupational groups, (MDs versus nurses versus the others), which showed that these three groups differ from each other (*p* = 0.015). A post hoc analysis (multiple comparisons) indicated a statistically significant difference between MDs and nurses in terms of risk-taking behavior (venturesomeness) (*p* = 0.012). The values of the venturesomeness variable for nurses (M = 4.4; Me = 3.0; SD = 3.64) were lower than those for medical doctors. There were no statistically significant differences in terms of IVE test results between the carriers of the long 7R allele and the others (Table 7). In addition, a negative Spearman’s rank correlation was observed between venturesomeness and age (−0.44, *p* < 0.05) and length of service in the profession (seniority) (rs = −0.46, *p* < 0.05).

### 2.3. Stress-Coping Techniques—MiniCOPE Results

Women are more likely to blame themselves (*p* = 0.039), while men are significantly more likely to cope with stress with humor (*p* = 0.016) (Table 8). Table 9 presents the descriptive and comparative statistics for the mini-COPE inventory in the study cohort by profession (MD versus non-MD). Medical doctors (MDs) were more likely to cope with stress with humor than other healthcare professionals (Table 9). *DRD4*-R7 allele carriers were significantly less likely to use stimulants and other substances to help themselves with stress coping (*p* = 0.0203) and less likely to expect emotional support (*p* = 0.0219) (Table 10).

The older and more experienced healthcare professionals were less likely to use stress management techniques such as use of emotional support and venting. In Spearman’s rank correlation analysis between the variable age and the variable use of emotional support, the result was rs = −0.308, *p* < 0.05. In Spearman’s rank correlation analysis between the variable age and the variable venting, the result rs = −0.224 *p* < 0.05 was obtained. In Spearman’s rank correlation analysis between the variable seniority and the variable use of emotional support, the result was rs = −0.284, *p* < 0.05. In Spearman’s rank correlation analysis between the variable seniority and the variable venting, the result was rs = −0.222 *p* < 0.05.

In Spearman’s rank correlation analysis between the age variable and the religion variable (as a stress-coping technique), the result was rs = 0.225, *p* < 0.05, indicating that older healthcare professionals became more religious. In Spearman’s rank correlation analysis between the variable seniority and the variable sense of humor, the result rs = −0.232, *p* < 0.05 was obtained.

Non-surgical professionals were more focused on the so-called problem-focused strategies, namely, active coping, planning and positive reframing. Multifactor models revealed the following relationships within the Mini-COPE inventory: active coping, by type of work position (β = 0.23; 95% CI: 0.05–0.42; *p* = 0.0121), planning, by type of work position (β = 0.19; 95% CI: 0.01–0.37; *p* = 0.0345), and positive reframing, by type of work position (β = 0.29; 95% CI: 0.04–0.53; *p* = 0.0221).

## 3. Discussion

Exposure to risky situations, including those in professional life, can affect risk tolerance. However, research also indicates that risk-coping attitudes are partly heritable [15]. Given the role of the dopaminergic system in reward and motivation mechanisms and impulse control [16], genes related to this neurotransmitter are potential candidates for studying the genetic basis of differential stress sensitivity and risk-taking propensity. High polymorphism, expression in the frontal cortex (an extremely important area in decision-making) and its large region of variable number of tandem repeats (VNTRs) make the *DRD4* gene a very good research target [17]. The GWAS study did not show a statistically significant genetic contribution of genes involved in the dopaminergic pathway to risk attitudes, but we know that such studies, by eliminating any environmental conditions or gene interactions, can mask some genetic factors [18].

Studies indicate, for instance, that the effect of the *DRD4* gene polymorphism can be modulated already at a very early, even prenatal, stage (e.g., gyrification ceases by about the age of two) [19,20,21]. *DRD4*-7R carriers with diagnosed ADHD have less prefrontal gyrification than individuals with the other *DRD4* allele, suggesting the *DRD4*-7R allele could be involved in this hypogyrification [19].

A study by Zohsel et al. (2014) [20] indicates an association between a mother’s stress during pregnancy and the propensity for aggressive behavior in her offspring, especially with the co-occurrence of adverse environmental factors. Carriers of at least one allele containing seven repeats (*DRD4*-7R), whose mothers indicated a state of increased stress during pregnancy using the questionnaire method, were found to be at increased risk for a diagnosis of conduct disorder and/or oppositional defiant disorder. Furthermore, homozygous carriers of the *DRD4*-7R allele showed more antisocial behavior after exposure to higher levels of prenatal maternal stress, while homozygous carriers of the DRD4 4R allele appeared insensitive to the effects of prenatal stress [20]. Bakermans-Kranenburg et al. (2011) [21] tested 124 adopted adults, showing that carriers of the *DRD4*-7R allele who had experienced parental problems had the highest scores for unresolved loss or trauma, while *DRD4*-7R carriers who had not experienced parental problems showed the lowest trauma scores. Importantly, among participants without the *DRD4*-7R allele, parental problems in childhood made no difference. This study supports the hypothesis for heightened susceptibility to environmental influences for carriers of the *DRD4*-7R allele instead of only increased vulnerability [21]. The findings provide independent evidence for interaction of *DRD4* 7R and maternal sensitivity affecting externalizing behavior in children, with carriers of the *DRD4*-7R allele having greater externalizing problems in the face of maternal insensitivity [21,22]. Zandstra and colleagues pointed out the significance of chronic multi-context stressors, showing that higher levels of chronic stressors were associated with higher levels of externalization in *DRD4*-7R allele carriers (high sensitivity to adverse environmental conditions), but not in non-carriers (low sensitivity) [23].

Many researchers also emphasize that some inconsistency in the data regarding *DRD4*-7R may reflect individual differences in sensitivity to environmental influences [24]. Clochard et al. (2023) [14] showed that the increase in risk tolerance due to the *DRD4*-7R allele was moderated in an additive matter (not dominant, as previously thought) and was not directly related to environmental risk. Their study was conducted on two populations of fishermen from small villages in northern Senegal, one of which was at very high risk due to unsafe fishing [14]. In addition, their data did not support the association of the *DRD4*-7R allele with novelty seeking, as previously found [14,24]. A meta-analysis of 20 studies reported that only 13 analyzed scientific studies showed a link between the presence of longer alleles (with more repeats, e.g., R7) and higher novelty-seeking scores [24]. The authors of the meta-analysis also pointed out the validity of searching for factors that moderate this relationship [24], which makes research on different populations even more important.

Armbruster and associates studied 84 healthy adult volunteers demonstrating the effect of the *DRD4* gene polymorphism on stress reactivity. They examined salivary cortisol levels during and after the Trier social stress test. Carriers of the *DRD4*-7R allele demonstrated lower cortisol responses [25]. This would suggest greater resistance to stress.

The dopamine receptor D4 is a dopamine D2-like G protein-coupled receptor encoded by the *DRD4* gene located on chromosome 11 at 11p15.5 [26].

Individuals with the *DRD4*-7R allele were found to have increased daytime sleepiness compared to those without the allele. However, it is not known whether this is due to the expression of the *DRD4* receptor in the retina, thus affecting circadian rhythms, or whether the increased sensitivity to the environment simply results in greater cognitive processing that causes fatigue [27,28,29].

Gehricke et al. performed an fMRI study on healthy volunteers, showing increased brain activity in response to unpleasant images compared to neutral images in the right temporal lobe in participants with the *DRD4*–4R/7R genotype versus participants with the *DRD4*–4R/4R genotype (without the long allele). This finding suggests greater involvement of the *DRD4*-7R allele in processing negative emotional stimuli [30].

Colzato et al. (2010) found that carriers of the *DRD4*-7R allele exhibited higher dysfunctional impulsivity (the tendency to act without thinking when such action is inappropriate) [31]. Several meta-analyses identified the *DRD4*-7R allele as a risk allele for attention deficit hyperactivity disorder (ADHD), a disorder characterized by high impulsivity [32,33]. In our study, carriers of the *DRD4*-7R allele were mainly medical doctors (physicians). This group was also characterized by greater venturesomeness (statistically significant difference) and impulsivity.

In our study, as many as 30 out of 82 participants (36,59%) were carriers of at least one long *DRD4*-7R allele (see Table 1 and Table 2 in the Section 2.1). The study group consisted of Caucasian healthcare workers employed in medical centers in central Poland. This is important because, although the 4R allele is the most common in all geographical regions [27], 7R alleles vary significantly in frequency across populations and ethnic groups. The 7R allele has low prevalence in Asia (2%), but high prevalence in America (48%) [29,34].

The present study indicates that *DRD4*-R7 allele carriers were significantly less likely to use stimulants and other substances to help themselves with stress coping. This contradicts previous reports that carriers of the *DRD4*-7R allele were more susceptible to addictions [35,36,37,38]. As previously mentioned, a longer allele of the *DRD4* gene resulted in lower dopamine receptor affinity; so, people with this version of the allele needed more dopamine for optimal well-being [39,40].

According to our study, carriers of the *DRD4*-7R allele are less likely to seek emotional support in stressful situations. This may be due to a greater tendency towards impulsive behavior, greater involvement in processing negative stimuli or stress resistance. The results of the study by Su et al. suggest that motion control serves as one modulator by which genetic predisposition and gene–environment interactions affect behavior [40]. Women who carried the long allele of the *DRD4* gene (genetic factor) and experienced more adverse life events (environmental factor) were less sensitive in interactions with their children [41]. Moreover, a study by Tompson et al. showed that East Asian carriers (compared to non-carriers) of the 7/2R *DRD4* allele reported experiencing greater emotional balance (i.e., a weaker positive attitude) than non-carriers of these alleles [42].

One of the main limitations of this study is the small sample size. Nevertheless, we believe that this study may contribute to further research on a larger scale.

## 4. Materials and Methods

### 4.1. Participants

The preliminary stage of this study involved 82 volunteers from among active healthcare professionals (66 females and 16 males), including 33 medical doctors (MDs). Other participants were occupationally active: nurses (*n* = 37), paramedics (*n* = 4), physiotherapists (*n* = 3), clinical psychologists (*n* = 4), and a midwife (*n* = 1). All participants had a direct contact with patients, which may constitute a risk factor for professional burnout. Approximately half of the participants (*n* = 40, 48.78%) were working in non-surgical medical specialties. All participants filled out the questionnaire regarding their social, family and occupational situation. Table 11 shows sociodemographic characteristics of the study group. Considering the predominance of female participants, an analysis of the cohort sociodemographic data by gender was also conducted (Table 12). The only statistically significant difference (*p* < 0.05) between genders was observed when comparing the type of work performed (surgical, non-surgical and emergency/IC—intensive care). The predominance of women is no coincidence. Women constitute a significant and growing proportion of Poland’s healthcare workforce, particularly in nursing [43].

### 4.2. Methods

The study participants were asked to fill out a questionnaire about their professional situation (including occupation, education, length of working-time in the profession (seniority), and type of work position: surgical, non-surgical or emergency/intensive care). Furthermore, they were asked to fill out psychological questionnaires for assessment of anxiety levels, coping effectiveness, risk-taking propensity and signs of burnout syndrome:

− **IVE—Impulsivity Questionnaire**—consists of 54 questions with Yes or No answers. The results are captured on three scales: Impulsivity, Willingness to Risk (venturesomeness) and Empathy (54 questions) [44,45].
− **Mini-COPE—Inventory for the Measurement of Coping with Stress**—consists of 28 statements included in 14 strategies (2 statements in each strategy). The method is most commonly used to measure dispositional coping, i.e., assessing typical ways of reacting and feeling in situations of experiencing severe stress (28 questions in total). Problem-focused coping strategies include active coping, planning, positive reframing and the use of instrumental support. Emotion-focused coping technics are acceptance, humor, religion, the use of emotional support, venting and self-blame. Avoidance coping covers self-distraction, denial, substance use and behavioral disengagement [46].

A cheek swab was collected from the study participants to determine the polymorphism within the gene for the dopamine D4 receptor gene (*DRD4*). Genomic DNA was isolated from nasal swabs by using the EXTRACTME swab & semen kit (Blirt, Gdansk, Poland) as per the protocol provided by the manufacturer. After evaluating the quantity and purity of the samples by measuring the ratio between absorbance at 260 nm and 280 nm on a Picodrop UV/Vis spectrophotometer, the samples were stored at −20 °C. To determine the distribution of *DRD4* polymorphisms, these specific primers were designed using Primer-BLAST: forward 5′-CGTACTGTGCGGCCTCAACGA-3′, reverse 5′-GACACAGCGCCTGCGTGATGT-3′. The product length was 705 bp (base pairs) for variant 4R, 609 bp for 2R and 849 bp for 7R, and after performing a gradient melting curve to validate the primers on the MJ Mini Thermal Cycler (Bio-Rad, Hercules, CA, USA), the proper PCR reaction was performed using PCR Mix Plus HGC (A&A Biotechnology, Gdansk, Poland). Each of the reactions included 10 ng of genomic DNA, PCR-grade water and PCR Mix containing Polymerase Taq, MgCl_2_ and dNTPs (Deoxynucleotide Triphosphates) provided in the kit. The following thermal conditions were applied: initial denaturation at 95 °C for 180 s, followed by 40 cycles consisting of 95 °C for 25 s, 45 s of annealing at 65 °C and 45 s at 72 °C, ending with an additional 10 min of extending at 72 °C. The products were subsequently separated on 2% agarose gel stained with SimplySafe (EURx, Gdansk, Poland) dye during an hour-long electrophoresis at 100 V, preceded by 5 min at 80 V to avoid smearing. Finally, visualization was performed on ImageMaster VDS (Pharmacia Biotech, Uppsala, Sweden).

The method of obtaining genetic material from a buccal swab was chosen because it is safe, non-invasive, simple, and quick. At the same time, it is assumed that the genetic makeup is the same across the body.

A statistical analysis was performed. Categorical traits were described through integer numbers and percentages. Numerical traits were depicted by using their mean, median, standard deviation, and lower-to-upper quartile values. The Chi-squared test of independence was performed for contingency tables with the required number of observations per cell. Otherwise, Fisher’s exact test was applied. The normality of distribution of a numerical trait was assessed by using the Shapiro–Wilk W test. Levene’s test was used in order to appraise the homogeneity of variances. The Mann–Whitney test was used when data was not normally distributed to compare two independent groups. In order to compare more than two independent groups, the Kruskal–Wallis non-parametric test was used. Spearman’s rank correlation coefficient was used to measure the dependence between two not-normally distributed variables. A best subset regression model was carried out in order to test the significance of differences in normally distributed numerical traits between selected study groups, controlling for important patients’ characteristics and confounders. Binary logistic regression models were performed for dichotomous dependent variables, whereas ordinal regression models were fitted for ordinal dependent variables. A level of *p* < 0.05 was deemed statistically significant. All the procedures were performed by using Statistica™, release 13 (TIBCO Software Inc., Palo Alto, CA, USA).

## 5. Conclusions

To sum up, our study indicates that *DRD4*-R7 allele carriers are less likely to use stimulants and other substances to help with stress coping and less likely to expect emotional support. The *DRD4* gene polymorphism may be important for the development of specific personality traits and ways of coping with stress. However, further research with larger numbers of participants is needed. It is particularly important to search for modulating factors, as research indicates the significant importance of gene–environment interactions.

## Figures and Tables

**Table 1:** Detailed genotypic characteristics of the study cohort.

*DRD4* Gene Polymorphism:	*n* (%)
2R2R	1 (1.22)
2R4R	19 (23.17)
2R7R	3 (3.66)
4R4R	32 (39.02)
4R7R	25 (30.49)
7R7R	2 (2.44)

**Table 2:** Genotypic characteristics of the study cohort.

*DRD4*-7R:	*n* (%)
- Present	30 (36.59)
- Absent	52 (63.41)
**Specific group:**	
- One 7R allele	28 (34.15)
- Two 7R alleles	2 (2.44)
- None	52 (63.41)

**Table 3:** Genotypic characteristics of the study cohort by type of profession (MD versus non-MD).

*DRD4*-7R:	MD *n* (%)	Non-MD *n* (%)	*p*-Value
- Present	18 (54.55)	12 (24.29)	0.0056
- Absent	15 (45.45)	37 (75.51)	

**Table 4:** Genotypic characteristics of the study cohort by type of profession (MD versus nurses versus others).

*DRD4*-7R:	MD *n* (%)	Nurse *n* (%)	Other *n* (%)	*p*-Value
- Present	18 (54.55)	9 (32.14)	3 (25.00)	0.0225
- Absent	15 (45.45)	28 (75.68%)	9 (75.00)	

**Table 5:** IVE test results of the study cohort by gender.

IVE	Female				Male				
	Statistical Parameter								
	M	SD	Me	Q_1_–Q_3_	M	SD	Me	Q_1_–Q_3_	*p*-Value
Impulsiveness	5.33	3.25	5.00	3.00–7.00	6.31	2.75	6.50	4.50–7.50	0.138
**Venturesomeness**	**4.85**	**3.62**	**4.00**	**2.00–8.00**	**8.50**	**3.48**	**9.50**	**5.50–11.00**	0.001 *
**Empathy**	**14.06**	**2.76**	**15.00**	**13.00–16.00**	**12.06**	**2.89**	**12.50**	**10.00–15.00**	0.008 *

* The bold character indicates significant (*p* < 0.05).

**Table 6:** IVE test results of the study cohort by type of profession (MD versus non-MD).

IVE	MD				Non-MD				
	Statistical Parameter								
	M	SD	Me	Q_1_–Q_3_	M	SD	Me	Q_1_–Q_3_	*p*-Value
Impulsiveness	6.09	3.03	5.00	4.00–9.00	5.14	3.23	5.00	3.00–7.00	0.111
**Venturesomeness**	**6.91**	**3.74**	**6.00**	**4.00–10.00**	**4.65**	**3.70**	**3.00**	**2.00–8.00**	0.005 *
Empathy	13.39	2.63	15.00	12.00–15.00	13.86	3.04	15.00	12.00–16.00	0.321

* The bold character indicates significant (*p* < 0.05).

**Table 7:** IVE test results of the study cohort by 7R allele presence.

IVE	7R Allele Presence				7R Allele Absence				
	Statistical Parameter								
	M	SD	Me	Q_1_–Q_3_	M	SD	Me	Q_1_–Q_3_	*p*-Value
Impulsiveness	4.93	3.40	4.50	3.00–7.00	5.87	3.00	5.50	4.00–8.00	0.137
Venturesomeness	6.00	4.20	5.00	2.00–10.00	5.31	3.66	5.00	3.00–8.50	0.511
Empathy	13.50	2.90	15.00	11.00–15.00	13.77	2.89	15.00	12.00–16.00	0.733

**Table 8:** Descriptive and comparative statistics for the mini-COPE inventory in the study cohort by gender.

Mini-COPE’s Strategies	Female				Male				
	Statistical Parameter								
	M	SD	Me	Q_1_–Q_3_	M	SD	Me	Q_1_–Q_3_	*p*-Value
Active coping	2.29	0.55	2.00	2.00–3.00	2.12	0.69	2.00	2.00–2.50	0.486
Planning	2.24	0.55	2.00	2.00–2.50	1.91	0.76	2.00	1.50–2.50	0.151
Positive reframing	1.84	0.67	2.00	1.50–2.50	1.81	0.83	2.00	1.75–2.25	0.764
Acceptance	1.83	0.56	2.00	1.50–2.00	1.91	0.76	2.00	1.75–2.25	0.412
**Humor**	**0.89**	**0.64**	**1.00**	**0.50–1.50**	**1.37**	**0.85**	**1.25**	**1.00–1.75**	0.016 *
Religion	1.05	0.98	1.00	0.00–1.50	0.53	0.67	0.25	0.00–1.00	0.051
Use of emotional support	2.03	0.71	2.00	1.50–2.50	1.62	0.92	1.75	1.00–2.25	0.092
Use of instrumental support	2.01	0.68	2.00	1.50–2.50	1.84	0.81	2.00	1.50–2.25	0.469
Self-distraction	1.70	0.67	1.75	1.00–2.00	1.62	0.69	1.50	1.25–2.00	0.737
Denial	0.63	0.69	0.50	0.00–1.00	0.56	0.79	0.25	0.00–1.00	0.551
Venting	1.44	0.58	1.50	1.00–2.00	1.28	0.60	1.25	1.00–1.50	0.304
Substance use	0.42	0.60	0.00	0.00–1.00	0.53	0.62	0.50	0.00–1.00	0.387
Behavioral disengagement	0.83	0.69	0.75	0.50–1.50	0.84	0.65	0.75	0.50–1.25	0.857
**Self-blame**	**1.51**	**0.76**	**1.50**	**1.00–2.00**	**1.06**	**0.75**	**1.00**	**0.50–1.75**	0.039 *

* The bold character indicates significant (*p* < 0.05).

**Table 9:** Descriptive and comparative statistics for the mini-COPE inventory in the study cohort by profession (MD versus non-MD).

Mini-COPE’s Strategies	MD				Non-MD				
	Statistical Parameter								
	M	SD	Me	Q_1_–Q_3_	M	SD	Me	Q_1_–Q_3_	*p*-Value
Active coping	2.33	0.57	2.50	2.00–3.00	2.21	0.59	2.00	2.00–2.50	0.390
Planning	2.29	0.47	2.00	2.00–2.50	2.10	0.68	2.00	2.00–2.50	0.284
Positive reframing	1.86	0.73	2.00	1.50–2.50	1.82	0.69	2.00	1.50–2.50	0.801
Acceptance	1.95	0.54	2.00	1.50–2.00	1.77	0.63	2.00	1.50–2.00	0.139
**Humor**	**1.23**	**0.80**	**1.00**	**0.50–1.50**	**0.82**	**0.59**	**1.00**	**0.50–1.00**	0.020 *
Religion	0.83	0.84	0.50	0.00–1.50	1.03	1.01	1.00	0.00–2.00	0.500
Use of emotional support	1.94	0.72	2.00	1.00–2.50	1.96	0.80	2.00	1.50–2.50	0.830
Use of instrumental support	1.97	0.72	2.00	1.50–2.50	1.99	0.70	2.00	1.50–2.50	0.907
Self-distraction	1.71	0.63	2.00	1.50–2.00	1.66	0.70	1.50	1.00–2.00	0.621
Denial	0.58	0.75	0.50	0.00–1.00	0.64	0.68	0.50	0.00–1.00	0.471
Venting	1.50	0.48	1.50	1.00–2.00	1.35	0.64	1.50	1.00–2.00	0.302
Substance use	0.52	0.64	0.60	0.00–1.00	0.40	0.58	0.00	0.00–1.00	0.338
Behavioral disengagement	0.68	0.61	0.50	0.00–1.00	0.93	0.71	1.00	0.50–1.50	0.151
Self-blame	1.53	0.86	1.50	1.00–2.00	1.36	0.72	1.50	1.00–2.00	0.521

* The bold character indicates significant (*p* < 0.05).

**Table 10:** Descriptive and comparative statistics for the mini-COPE inventory in the study cohort by occurrence of the 7R allele.

Mini-COPE’s Strategies	7R Allele Present				7R Allele Absent				
	Statistical Parameter								
	M	SD	Me	Q_1_–Q_3_	M	SD	Me	Q_1_–Q_3_	*p*-Value
Active coping	2.32	0.52	2.00	2.00–3.00	2.23	0.61	2.00	2.00–2.75	0.6224
Planning	2.32	0.52	2.50	2.00–2.50	2.10	0.64	2.00	2.00–2.50	0.1131
Positive reframing	1.80	0.76	2.00	1.00–2.00	1.86	0.67	2.00	1.50–2.50	0.7039
Acceptance	1.97	0.61	2.00	1.50–2.50	1.77	0.58	2.00	1.50–2.00	0.2286
Humor	0.95	0.77	0.75	0.50–1.00	1.00	0.68	1.00	0.50–1.50	0.3904
Religion	0.78	0.94	0.50	0.00–1.50	1.05	0.94	1.00	0.00–2.00	0.1547
**Use of emotional support**	**1.68**	**0.74**	**2.00**	**1.00–2.00**	**2.11**	**0.74**	**2.00**	**2.00–2.50**	0.0219 *
Use of instrumental support	1.82	0.78	2.00	1.00–2.50	2.08	0.64	2.00	1.75–2.50	0.1689
Self-distraction	1.67	0.70	1.75	1.00–2.00	1.69	0.66	1.50	1.00–2.00	0.8743
Denial	0.57	0.73	0.50	0.00–1.00	0.64	0.70	0.50	0.00–1.00	0.5268
Venting	1.42	0.54	1.50	1.00–2.00	1.40	0.61	1.25	1.00–2.00	0.8996
**Substance use**	**0.30**	**0.53**	**0.00**	**0.00–0.50**	**0.53**	**0.63**	**0.25**	**0.00–1.00**	0.0203 *
Behavioral disengagement	0.83	0.62	0.75	0.50–1.00	0.83	0.72	0.50	0.25–1.50	0.2618
Self-blame	1.58	0.76	1.50	1.00–2.00	1.34	0.78	1.00	1.00–2.00	0.1843

* The bold character indicates significant (*p* < 0.05).

**Table 11:** Sociodemographic characteristics of the study group (discrete variables) (*n* = 82 individuals).

Analyzed Trait	*n* (%)
Gender:	
- Female	66 (80.49)
- Male	16 (19.51)
Age group (years):	
- Up to 35	27 (32.93)
- 36–50	34 (41.46)
- 51+	21 (25.61)
Profession:	
- Medical doctor (MD)	33 (40.24)
- Other medical professions	49 (59.76)
Place of residence:	
- City (over 500 k)	56 (68.29)
- City (150–500 k)	5 (6.10)
- City (50–150 k)	5 (6.10)
- City (up to 50 k)	10 (12.20)
- Village	6 (7.31)
Marital status:	
- Married	51 (62.19)
- Single	13 (13.85)
In an informal relationship:	7 (8.54)
- Divorced	6 (7.32)
- Widowed	5 (6.10)
No. of children:	
- None	23 (28.40)
- 1	19 (23.46)
- 2	27 (33.33)
- 3	11 (13.58)
- 4	1 (1.23)
Seniority (years) *:	
- Up to 10	26 (32.10)
- 11–20	18 (22.22)
- 21–30	12 (14.82)
- 31+	25 (30.86)
Type of work position:	
- Surgical	29 (35.37)
- Non-surgical	40 (48.78)
- Emergency and IC	13 (15.85)
Professional degree:	
- Habilitated doctor	3 (3.71)
- PhD	16 (19.75)
- Master’s degree or equiv.	37 (45.68)
- Bachelor’s degree	9 (11.11)
- Secondary school	16 (19.75)
No. of work places:	
- 1	32 (39.02)
- 2	31 (37.81)
- 3	10 (12.19)
- 4	7 (8.54)
- 5	1 (1.22)
- 6	1 (1.22)
Medical on-call:	61 (74.39)
No. of work place changes:	
- None	34 (43.04)
- 1	17 (21.52)
- 2	16 (20.25)
- 3	7 (8.86)
- 4	2 (2.53)
- 5	0 (0.00)
- 6	3 (3.80)

* years of working in the profession.

**Table 12:** Sociodemographic characteristics of the study cohort by gender.

Analyzed Trait	Female	Male	*p*-Value
	*n* (%)	*n* (%)	
Age group (years):			0.2233
Up to 35	20 (30.30)	7 (43.75)	
36–50	16 (24.24)	5 (31.25)	
51+	20 (45.46)	4 (25.00)	
Medical doctor?			0.1456
Yes, MD	24 (36.36)	9 (56.25)	
No, other	42 (63.64)	7 (43.755)	
Seniority (years):			0.3434
Up to 10	19 (29.23)	7 (43.75)	
11–20	13 (20.00)	5 (31.25)	
21–30	11 (16.92)	1 (6.25)	
31+	22 (33.85)	3 (18.75)	
Type of work position:			0.0023 *
Surgical	**24 (36.36)**	**5 (31.25)**	
Non-surgical	**36 (54.55)**	**4 (25.00)**	
Emergency and IC	**6 (9.09)**	**7 (43.75)**	

* The bold character indicates significant (*p* < 0.05).

## Data Availability

The raw data supporting the conclusions of this article will be made available by the authors on request.
